# Effects of the SUMO Ligase BCA2 on Metabolic Activity, Cell Proliferation, Cell Migration, Cell Cycle, and the Regulation of NF-κB and IRF1 in Different Breast Epithelial Cellular Contexts

**DOI:** 10.3389/fcell.2021.711481

**Published:** 2021-09-13

**Authors:** Yuhang Shi, Sergio Castro-Gonzalez, Yuexuan Chen, Ruth Serra-Moreno

**Affiliations:** ^1^Microbiology and Immunology, University of Rochester Medical Center, Rochester, NY, United States; ^2^Department of Chemistry, Umeå University, Umeå, Sweden

**Keywords:** BCA2, antiviral defense, NF-κB, IRF1, cancer

## Abstract

Breast cancer-associated gene 2 (BCA2) is an E3 ubiquitin and SUMO ligase with antiviral properties against HIV. Specifically, BCA2 (i) enhances the restriction imposed by BST2/Tetherin, impeding viral release; (ii) promotes the ubiquitination and degradation of the HIV protein Gag, limiting virion production; (iii) down-regulates NF-κB, which is necessary for HIV RNA synthesis; and (iv) activates the innate transcription factor IRF1. Due to its antiviral properties, ectopic expression of BCA2 in infected cells represents a promising therapeutic approach against HIV infection. However, BCA2 up-regulation is often observed in breast tumors. To date, the studies about BCA2 and cancer development are controversial, stating both pro- and anti-oncogenic roles. Here, we investigated the impact of BCA2 on cellular metabolic activity, cell proliferation, cell migration, and cell cycle progression. In addition, we also examined the ability of BCA2 to regulate NF-κB and IRF1 in transformed and non-tumor breast epithelial environments. Despite the fact that BCA2 promotes the transition from G1 to S phase of the cell cycle, it did not increase cell proliferation, migration nor metabolic activity. As expected, BCA2 maintains its enzymatic function at inhibiting NF-κB in different breast cancer cell lines. However, the effect of BCA2 on IRF1 differs depending on the cellular context. Specifically, BCA2 activates IRF1 in ER^+^ breast cell lines while it inhibits this transcription factor in ER^–^ breast cancer cells. We hypothesize that the distinct actions of BCA2 over IRF1 may explain, at least in part, the different proposed roles for BCA2 in these cancers.

## Introduction

Breast cancer-associated gene 2 (BCA2, also known as Rabring7, RNF115 or ZNF364) is a RING-finger E3 ubiquitin and SUMO ligase with antiviral properties against human immunodeficiency virus (HIV) through different mechanisms. First, BCA2 is a co-factor of BST2/Tetherin, a well-studied restriction factor that traps nascent HIV virions to the cell membrane to hinder virion release (Neil et al., 2008; Van Damme et al., 2008). Specifically, BCA2 interacts with BST2/Tetherin, causing the internalization and lysosomal degradation of BST2-captured viral particles (Miyakawa et al., 2009). In addition to virus egress, BCA2 also impairs virus assembly by promoting the ubiquitination and lysosomal degradation of HIV Gag (Nityanandam and Serra-Moreno, 2014), the major structural protein for this virus. Furthermore, BCA2 prevents HIV transcription via negatively regulating NF-κB (Colomer-Lluch and Serra-Moreno, 2017), a critical transcription factor that facilitates HIV RNA synthesis (Nabel and Baltimore, 1987). In particular, BCA2 works as an E3 SUMO ligase by promoting the SUMOylation of IκBα, an inhibitor of NF-κB (Colomer-Lluch and Serra-Moreno, 2017). Under conditions of infection or inflammation, IκBα is ubiquitinated, which leads to its degradation, allowing in turn the activation and nuclear translocation of NF-κB. However, the SUMOylation of IκBα prevents its ubiquitination, enhancing even further its inhibitory effect over NF-κB (Wulczyn et al., 1996; Hayden and Ghosh, 2004). Due to all these antiviral roles, BCA2 is considered a potent host antiviral factor that poses several levels of restriction against HIV: at the transcription, assembly, and release levels.

Despite its antiviral properties, BCA2 has been associated with breast cancer development, since this protein is found overexpressed in over 50% invasive breast cancers, and its up-regulation correlates with estrogen receptor positive (ER^+^) status and poor prognosis (Burger et al., 2005, 2010). In addition to their aggressiveness, breast cancers are classified depending on the presence of markers such as the receptors for the hormones progesterone (PR) and estrogen (ER). Of note, ER^+^ and/or PR^+^ cancers have been associated with lower risks of mortality because of their better response to endocrine therapy (Costa et al., 2002; Bardou et al., 2003; Dunnwald et al., 2007). The fact that BCA2 expression correlates with ER status and disease outcome prompted several groups to investigate the role of BCA2 in cellular transformation. However, these studies have led to contradicting reports, stating both oncogenic and tumor suppressive roles for this protein. On one hand, some studies suggest that BCA2 plays oncogenic roles because (i) there is a strong link between BCA2 overexpression and tumorigenesis (Burger et al., 2005), and (ii) BCA2 causes the ubiquitination and proteasomal degradation of the tumor suppressor p21 (Wang et al., 2013). p21 is a well-characterized cyclin-dependent kinase (CDK) inhibitor that blocks the activity of cyclin/CDK2 complexes and causes cell cycle arrest (Abbas and Dutta, 2009; Karimian et al., 2016; Barr et al., 2017). Hence, the BCA2-mediated degradation of p21 would be in line with an oncogenic role for BCA2. On the other hand, other reports point toward a tumor suppressive role for BCA2 due to its effects on the down-regulation of the proto-oncogenes epidermal growth factor receptor (EGFR) and c-Myc (Narita et al., 2012; Smith et al., 2013; Wymant et al., 2016). EGFR induces cell differentiation and proliferation upon activation and has been found hyperactivated in many cancers due to the accumulation of mutations. BCA2 down-regulates EGFR by interacting with Rab7, a molecule involved in vesicular trafficking, to facilitate EGFR sorting to the lysosome for degradation (Smith et al., 2013; Wymant et al., 2016). c-Myc is a transcription factor that regulates entry in cell cycle and cell growth, and it is frequently dysregulated in many cancers. BCA2 has been reported to down-regulate c-Myc by promoting its ubiquitination and proteasomal degradation (Narita et al., 2012). In addition to these reports, studies derived from our group revealed that BCA2 prevents NF-κB activation, a transcription factor that not only plays a role in HIV transcription but also in cell survival and proliferation. In fact, NF-κB is normally found hyperactivated in many tumors (Biswas et al., 2004; Hoesel and Schmid, 2013; Li et al., 2013; Xia et al., 2014; Frasor et al., 2015). In addition to down-regulating NF-κB, we uncovered that BCA2 activates interferon regulatory factor 1 (IRF1) (Colomer-Lluch and Serra-Moreno, 2017), a well-known tumor suppressor and immunomodulatory transcription factor (Bouker et al., 2005), although the mechanism by which BCA2 achieves this remains to be elucidated. Hence, the impact of BCA2 on NF-κB and IRF1, together with its roles in promoting the degradation of EGFR and c-Myc, suggests that BCA2 may play a tumor suppressive role. Since the effect of BCA2 on cancer development has mainly been studied by looking at individual molecules that are targeted by BCA2, we decided to assess how this protein affects cell proliferation, migration, cell cycle progression, cellular metabolism as well as NF-κB and IRF1 status in a more general context. Understanding the impact of BCA2 on these cellular processes is critical to assess whether BCA2 can be a potential treatment for HIV infection.

Here, we show that despite promoting the transition from G1 to S phase of the cell cycle, BCA2 does not increase cellular proliferation, migration nor accelerates metabolic activity, and that its ability to regulate NF-κB is preserved for the most part. However, we found opposite roles for BCA2 in the modulation of IRF1. Whereas up-regulation of BCA2 activates IRF1 in ER^+^ breast cancer cell lines, the opposite effect was observed for ER^–^ and non-tumor breast cells. We hypothesize that these contrasting phenotypes may explain why the role of BCA2 in cancer formation has been controversial. In an effort to elucidate why BCA2 performs opposite roles in the regulation of IRF1, we performed mechanistic studies that revealed that in ER^+^ cell lines BCA2 physically interacts with IRF1 to enhance its translocation to the nucleus, which consequently would facilitate the expression of anti-tumor effectors. However, the ability of BCA2 to interact with IRF1 seems to be lost in more aggressive cancer cell lines like the ER^–^. Although this activity does not require BCA2’s enzymatic actions, loss of BCA2-IRF1 binding may contribute to more aggressive cancer development and might be considered as a biomarker for cancer prognosis.

## Materials and Methods

### Plasmid DNA Constructs

(1) BCA2 expression constructs. Human BCA2 (304 amino acids) and its mutants, C_22__8_AC_231_A, △Ring (a mutant that contains only amino acids 1-227), △C-GST (a mutant that contains only amino acids 1-147), △BZF (a mutant that contains only amino acids 47-304), and GST were cloned into expression vector pcDNA5. An HA-tag was added to their N-terminus, as previously described (Miyakawa et al., 2009; Nityanandam and Serra-Moreno, 2014; Colomer-Lluch and Serra-Moreno, 2017).
(2) Plasmids for the generation of virus-like particles (VLPs). psPAX2 packaging plasmid and pMD2-G envelope expressing vector were a gift from Dr. David T. Evans (University of Wisconsin, Madison WI). Vectors harboring shRNAs targeting BCA2 (shBCA2) and scrambled RNAs (shScramble) were obtained from the Thermo Fisher Scientific TRC consortium (Broad Institute, MIT and Harvard), as detailed previously (Nityanandam and Serra-Moreno, 2014).
(3) IRF1 expression construct. The expression vector pCMV6-IRF1 harbors a Myc and flag-tagged human *IRF1* gene. This construct was obtained from OriGene, Rockville, MD.
(4) NF-κB/IRF1 luciferase reporters and β-galactosidase vector. The NF-κB luciferase reporter and the β-galactosidase plasmid were a gift from Ronald C. Desrosiers (University of Miami, Miami, FL) (Postler and Desrosiers, 2012). The IRF1 luciferase reporter gene was obtained through Affymetrix, Santa Clara. CA.

### Cells and Transfections

Human HEK293T [American Type Culture Collection (ATCC), CRL-11268], MCF-7 (ATCC, HTB-22), and MDA-MB-231 (ATCC, HTB-26) cells were cultured in Dulbecco’s Modified Eagle Medium (DMEM, Thermo Fisher Scientific, 11885-084) supplemented with 10% fetal bovine serum (FBS, Thermo Fisher Scientific, 26140-079), 1% Penicillin-Streptomycin (Thermo Fisher Scientific, 15070-063) and 1% L-glutamine (Thermo Fisher Scientific, 25030-081). Human MCF-12F (ATCC, CRL-10783) cells were maintained in HuMEC medium (Thermo Fisher Scientific, 12753018) with HuMEC supplement kit (Thermo Fisher Scientific, 12755013). These cells were transfected using GenJet *in vitro* DNA transfection reagent (SignaGen Laboratories, SL100488) following the manufacturer’s instructions. Viability of cells was measured after each transfection. Only cells with viabilities of 90% and above were considered for further experiments.

### Knockdown of Endogenous BCA2

BCA2 depletion was achieved by transduction of viral-like particles harboring shScramble or shBCA2.

#### Viral-Like Particle (VLP) Generation

5x 10^6^ HEK293T cells were transfected with 3.75 μg psPAX2 packaging plasmid, 1.25 μg pMD2-G envelope expressing plasmid, and 5 μg shScramble RNA or shBCA2 containing plasmids. The supernatant was collected 48 h post-transfection and centrifuged for 10 min at 931 *× g* to remove cell debris. The concentration of VLPs was measured by p24 antigen-capture ELISA (Advanced Bioscience laboratories, 5421) following the manufacturer’s instructions.

#### Transduction

5 *×* 10^6^ of HEK293T, MCF-7, MDA-MB-231, and MCF-12F cells were seeded in 25 cm^2^ flasks. Twenty-four hours later, the cells were transduced with different combination of VLPs. Forty-eight hours later, cells were transduced with a second round of VLPs. One day after this second transduction, the cell medium was replaced and supplemented with puromycin (Thermo Fisher Scientific, A11138-03). Cells were cultured under puromycin for 10 days to allow for the selection of cells successfully transduced with shScramble or shBCA2. BCA2 knockdown was verified by RT-qPCR.

### XTT Assays

To assess for differences in cellular metabolism, the colorimetric assay, XTT [2,3-bis-(2-methoxy-4-nitro-5-sulfophenyl)-2H-tetrazolium-5-carboxanilide; Sigma-Aldrich, 11465015001] was performed, and an absorbance-based microplate reader (BMG LABTECH, LUMIstar Omega) was used to monitor fluctuations in metabolism.

6 *×* 10^5^ cells, including HEK293T, MCF-7, MDA-MB-231, and MCF-12F cells were seeded in 6-well plates, and transfected 24 h later with 2 μg of either pcDNA5, pcDNA5-HA-BCA2, or pcDNA5-HA-C_22__8_C_231_. Four hours post-transfection, the cell medium was replaced, and the cells were then re-seeded in 96-well plates at a density of 7,000 cells per well. The cells were incubated with the XTT reagents for 2 h before reading the assay. This experiment was performed multiple times to allow measurements at different time intervals. The total time points collected were 2, 24, 48, 72, 96, and 108 h after re-plating the cells. Absorbance at 475 and 650 nm was measured for each replicate and time interval. Metabolic readout was normalized by subtracting the absorbance measured at 650 nm from that obtained at 465 nm (475–650 nm). To examine the effects of endogenous BCA2 on cell metabolism, similar experiments were performed on cells transduced with shScramble and shBCA2. Briefly, stably transduced cells were seeded in 96-well plates at 7,000 cells per well. 2, 24, 48, 72, 96, and 108 h after seeding the cells, they were incubated with the XTT reagents for 2 h. The metabolic readout was calculated as explained above. For each experiment, parental cells and cells growing with 1% of serum (starvation control) were included. Each experiment was repeated three independent times and measured in duplicates.

### Cell Proliferation Assays

#### Cells Overexpressing HA-BCA2

5 *×* 10^4^ cells, including HEK293T, MCF-7, MDA-MB-231, and MCF-12F cells were seeded in 24-well plates and 24 h later they were transfected with 0.5 μg of pcDNA5, pcDNA5-HA-BCA2, or pcDNA5-HA-C_22__8_C_231_. Cell growth was measured by the number of cells accumulated 4, 24, 48, 72, and 96 h after transfection, and expressed as cell count over time.

#### Cells Depleted of BCA2

5 *×* 10^4^ shScramble-treated cells and stably BCA2-knocked down cells were seeded in 24-well plates. The effect of the depletion of BCA2 on cell growth was assessed by determining the cell number at 24, 48, 72, 96, and 108 h after the cells were seeded.

As for the XTT assays, parental cells and starvation treatment were included for each experimental scenario. All treatments were performed 3 independent times and measured in duplicates.

### Cell Migration Assays

Cells, including MCF-7, MDA-MB-231, and MCF-12F cells were plated in 6-well plates at a density of 1.2 *×* 10^6^ cells per well. Twenty-four hours later, cells were transfected with 3,000 ng of HA-GST or HA-BCA2. Similar assays were performed with cells stably transduced with a scrambled shRNA or shBCA2. BCA2 knockdown was verified by RT-qPCR on the day the cells were plated. When cells reached over 90% confluence, normally 24 h post-transfection, a scratch was performed with a micropipette tip on the monolayer. Next, the width and cell confluence of the wound was measured using a BioTek Lionheart automated microscope (BioTek, Winooski, VT). The closure of the wound was determined by live cell imaging over the course of 48 h.

### Cell Cycle Distribution Assays

3 *×* 10^5^ of HEK293T, MCF-7, MDA-MB-231, and MCF-12F cells were seeded in 6-well plates and transfected with 2 μg HA-GST or HA-BCA2. Forty-eight hours post-transfection, the cells were collected by adding trypsin-EDTA solution (Thermo Fisher Scientific, 25200056). Cells were permeabilized using FIX and PERM cell permeabilization kit (Thermo Fisher Scientific, GAS003), following the manufacturer’s instructions. Next, cells were stained with an anti-HA primary antibody (refer to Table 1 for a full list of antibodies and conditions) at a 1:200 ratio for 20 min at room temperature. After staining, cells were washed once with PBS at 500 *× g* for 5 min. Next, cells were incubated with a secondary antibody anti-mouse IgG_1_ Alexa Fluor 488 (Table 1) at a 1:500 ratio for 20 min at room temperature. After the secondary antibody, cells were washed with PBS, as detailed above. The cells were then stained with 7-AAD (Thermo Fisher Scientific, A1310) at a 1:400 dilution and incubated for 20 min at room temperature. Finally, cells were washed and fixed with 2% paraformaldehyde (Sigma-Aldrich, P6148). Data was collected on a BD Accuri C6 Plus Flow Cytometer (BD Biosciences, Franklin Lakes, NJ) and analyzed by FlowJo (version 10.7.1).

**TABLE 1:** Antibody sources and conditions.

Protein/tag	Antibody	Dilution	Source
HA	Mouse monoclonal to HA tag	1:1,000	BioLegend, 901502
IRF1	Rabbit monoclonal to IRF1	1:1,000	Cell signaling technology, 8478S
NF-κB p65	Rabbit monoclonal to NF-κB p65	1:1,000	Abcam, ab32536
UbcH5 (UBE2D1)	Mouse monoclonal to UBE2D1	1:1,000	Abcam, ab176561
Lamin A/C	Mouse monoclonal to Lamin A + Lamin C	1:1,000	Abcam, ab8984
BCA2 (RNF115)	Rabbit monoclonal to RNF115	1:500	Abcam, ab80432
β-actin	Mouse monoclonal to β-actin	1:1,000	MilliporeSigma, MAB1501
Myc	Mouse monoclonal to Myc tag	1:1,000	Abcam, ab18185
Anti-mouse IgG1	Goat polyclonal (HRP-conjugated)	1:4,000	Pierce, 31430
Anti-rabbit IgG	Donkey polyclonal (HRP-conjugated)	1:4,000	Abcam, ab16284
Anti-Goat IgG	Donkey polyclonal (HRP-conjugated)	1:6,000	Abcam, ab6885
Alexa-Fluor 488 IgG1	Mouse polyclonal (Alexa Fluor 488-conjugated)	1:500	Thermo Fisher Scientific, A21121

For BCA2 knocked down cell lines, 3 *×* 10^5^ cells were seeded and collected 48 h later. The cells were then centrifuged at 500 *× g* for 5 min and washed with PBS followed by staining with 7-AAD. Cells were processed and analyzed as detailed above. Each experiment was repeated three independent times.

### NF-κB and IRF1 Luciferase Reporter Assays

To assess the basal activation levels of NF-κB and IRF1, 3 *×* 10^5^ cells, including HEK293T cells, MCF-7, MDA-MB-231, and MCF-12F were seeded in 6-well plates and 24 h later transfected with 0.5 μg luciferase reporter gene (under the control of NF-κB or IRF1) and 0.05 μg of the β-galactosidase construct, which was used to normalize the variations in transfection efficiencies. Forty-eight hours post-transfection, the cells were washed with ice-cold Dulbecco’s phosphate-buffered saline (DPBS, Thermo Fisher Scientific, 14190-144) and lysed in reporter lysis buffer (Promega, Madison, WI) for 15 min at room temperature. The cell lysates were collected and centrifuged for 8 min at 16,000 *× g* at 4°C to remove debris. The supernatants were used to quantify luciferase activity (luciferase assay system; Promega, Madison, WI) and β-galactosidase activity (Galaco-Light Plus assay system; Applied Biosystems, Carlsbad, CA), which was measured by luminescence on a plate reader (BMG LABTECH, LUMIstar Omega), according to the respective manufacturer’s instructions. Luciferase activity was normalized to β-galactosidase activity and expressed as fold of NF-κB or IRF1 activation. The same protocol was applied for assays in which cells were depleted of BCA2 or transduced with shScramble.

For the HA-BCA2 overexpression and BCA2 mutant studies, similar assays were performed. However, the transfection conditions were somewhat different. For this, we used a combination of 0.5 μg luciferase reporter vector (NF-κB or IRF1), 1 μg HA-BCA2 plasmid (empty vector, C_22__8_C_231_, ΔBZF, ΔC-GST, or GST), and 0.05 μg of the β-galactosidase construct. Thirty-six hours post-transfection, the cells were incubated with DMSO or 10 ng/mL TNFα or PMA (phorbol myristate acetate). Twelve hours later, the cells were lysed, and luciferase activity was measured as describe above. Each experiment was repeated three independent times and measured in duplicates.

### RT-qPCR Assays

#### RNA Extraction and cDNA Synthesis

Cells, including HEK293T, MCF-7, MDA-MB-231, and MCF-12F were transfected with mock, pcDNA5, or pcDNA-5-HA-BCA2. Forty-eight hours later, cells were detached and washed with DPBS and total RNA was extracted using Qiagen RNeasy minikit (74004), following the manufacturer’s instructions. The same protocol of RNA extraction was applied to cells stably depleted of BCA2 or transduced with shScramble. RNA concentration and integrity were measured by a NanoDrop spectrophotometer and a BioAnalyzer, respectively. Only samples with RIN values above 8 were considered suitable for downstream analyses. Next, 1 μg of purified RNA was reverse transcribed and converted into cDNA using the iScript cDNA synthesis kit (Bio-Rad, 1725037) following the manufacturer’s instructions.

#### RT-qPCR

For each sample, different controls including RNA quality (RQ1 and RQ2), genomic DNA contamination (gDNA), and housekeeping gene (GAPDH) were measured by qPCR. In each PCR reaction, 10 μL 2x SsoAdvanced universal SYBR green supermix (Bio-Rad, 1725272), 0.2 μL cDNA, 8.8 μL RNase free water (Thermo Fisher Scientific, 10977-015), and 1 μL primer pair for the target gene or control were included. As target genes, primers specific for *BCA2 (RNF115)* and *IRF1* (Bio-Rad, PrimePCR) were included to assess for differences in their relative expression levels. The amplification program was as follows: 2 min at 95°C for initial activation, 40 cycles at 95°C for 5 s, 60°C for 30 s, and then melting analyses from 65 to 95°C (0.5°C increments). Each sample was analyzed by qPCR in two technical replicates. Experiments were performed three independent times for each experimental condition. All primers for control and target were obtained from Bio-Rad, Hercules, CA (RQ1 and RQ2: 10025694, gDNA: qHsaCtlD0001004, GAPDH: qHsaCED0038674, IRF1: qHsaCED0044080, RNF115: qHsaCID0017334).

### Subcellular Fractionation Assays

5 × 10^6^ cells, including HEK293T, MCF-7, MDA-MB-231, and MCF-12F were lysed using the ProteoExtract subcellular proteome extraction kit (S-PEK) (Millipore, 539790), following the manufacturer’s instructions. In particular, cytosolic fraction (F1) and nuclear fraction (F3) were evaluated by western blotting to measure the levels of BCA2, NF-κB (RELA/P65), IRF1, Lamin A/C, UbcH5, and β-actin using specific antibodies (Table 1). Lamin A/C and UbcH5 were used to determine the purity of nuclear and cytoplasmic fraction, respectively. β-actin was used as a loading control. Each experiment was repeated three independent times.

### Western Blotting

Cells, including HEK293T, MCF-7, MDA-MB-231, and MCF-12F with different treatments were washed with DPBS and incubated on lysis IP buffer (Thermo Fisher Scientific, 87787) on ice for 30 min. Cell debris was removed by centrifugation at 16,000 *× g* at 4°C for 8 min. The supernatants were obtained and mixed with 2x SDS sample buffer (Sigma-Aldrich, S3401), samples were then boiled for 5 min on a heat block. Next, proteins were separated using SDS-PAGE polyacrylamide gels (8–12%). Proteins were transferred to a polyvinylidene difluoride (PVDF) membrane (BioRad, 1620177) using a Trans-Blot SD transfer cell (BioRad, 1703940). Membranes were incubated for 1 h with blocking buffer (BioRad, 1706404) at room temperature, followed by an overnight incubation with primary antibodies (Table 1) at 4°C. Next, membranes were washed 3 times with PBS-tween (Sigma-Aldrich, P3563) followed by a 1-h incubation with the secondary antibodies (Table 1) at room temperature. Subsequently, three additional washes in PBS-tween were performed before imaging the membranes. Finally, membranes were developed by adding SuperSignal West Femto maximum sensitivity substrate (Pierce, 34095), and proteins were visualized in a Li-Cor Odyssey Fc Imager 2800 (Li-Cor, Lincoln, NE) and a ChemiDoc imaging system (BioRad, Hercules, CA).

### Co-immunoprecipitation Assays

Cells, including HEK293T, MCF-7, MDA-MB-231, and MCF-12F with different treatments were washed with DPBS and incubated on lysis IP buffer (Thermo Fisher Scientific, 87787) supplemented with protease inhibitors (Roche, 04693116001) and phosphatase inhibitor cocktails 2 and 3 (Sigma-Aldrich, P5726 and P0044) on ice for 1 h. After pre-clearing the cell lysates, samples were incubated with protein G magnetic beads (New England Biolabs, S1430S) for 1 h at room temperature to remove any unspecific binding. At the same time, fresh protein G beads were coated with the antibody of interest (anti-IRF; Table 1) for 1 h at room temperature, followed by three washes with coupling buffer (Thermo Fisher Scientific, 88805) to remove excess antibody. Next, the pre-cleared lysates were incubated with the antibody-coated protein G beads overnight at 4°C. The following day, beads were washed with lysis IP buffer 4 times using a magnetic rack. Finally, the beads were resuspended in 2x SDS sample buffer and the samples were analyzed by western blotting. As controls, we included samples consisting of IP lysis buffer mixed with beads and antibody (IgG control). These controls helped rule out any unspecific bands detected by western blot that corresponded to the IgG heavy or light chains or material from the magnetic beads.

### Statistical Analysis

Statistical calculations were performed using ANOVA with *post hoc* analyses, except for the assays to verify BCA2 knockdown. In this case, a two-tailored unpaired Student *t*-test analysis was used. All statistical analyses were performed using Graph Pad Prism version 9.1.2. *p*-values ≤ 0.05 were considered statistically significant.

## Results

### BCA2 Does Not Increase Cellular Metabolic Activity

**FIGURE 1:**
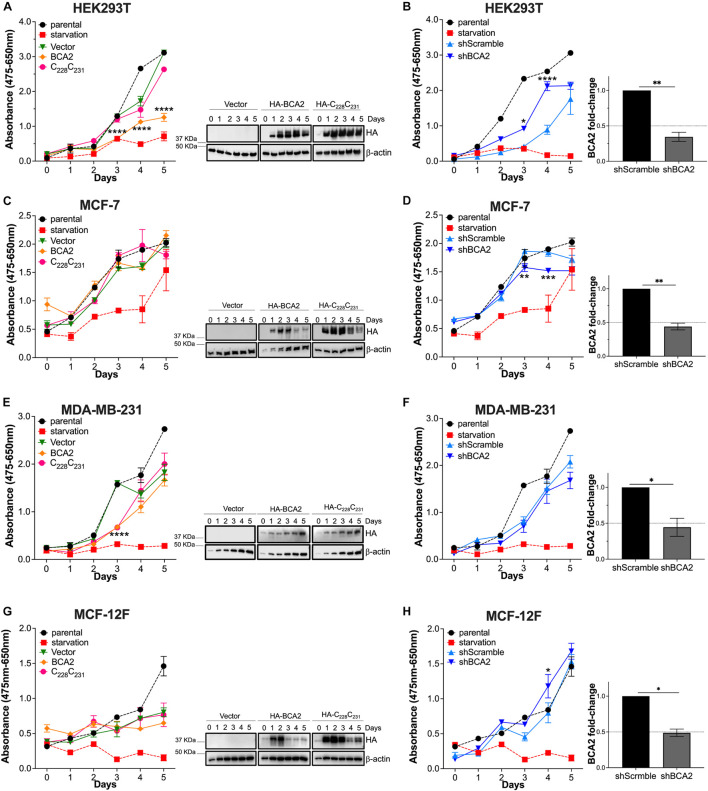
Effects of BCA2 on metabolic activity. **(A,C,E,G)** Cells, including HEK293T cells **(A)**, MCF-7 **(C)**, MDA-MB-231 **(E)**, and MCF-12F **(G)** were transfected with constructs encoding for HA-BCA2, a BCA2 catalytic-defective mutant (HA-C_22__8_C_231_) or an empty vector control. Cellular metabolic activity was measured over time by XTT assays. As controls, parental cells and cells under nutrient deprivation were included. Representative western blots for the expression of HA-BCA2 and HA-C_22__8_C_231_ are shown next to the graphs. **(B,D,F,H)** Similar experiments were performed in cells stably transduced with a scrambled shRNA or depleted of BCA2. BCA2 depletion was verified on the first and last day of analysis by RT-qPCR (right panels show BCA2 levels on day 5 of the assay). Dotted lines represent threshold for biological significance. Data correspond to the mean and SEM of 3 independent experiments. *p < 0.05, **p < 0.01, ***p < 0.001, ****p < 0.0001.

To understand the overall effect of BCA2 on tumor development and growth, we first evaluated the impact of BCA2 overexpression and depletion on cellular metabolic activity by using the XTT assay. This assay evaluates the conversion of XTT, 2,3-bis-(2-methoxy-4-nitro-5-sulfophenyl)-2H-tetrazolium-5-carboxanilide to water-soluble orange-colored formazan by nicotinamide adenine dinucleotide (NADH) in the mitochondria, and serves as an indirect measure of events that may affect metabolic function such as cell viability, proliferation and cytotoxicity. For this, we used the following cell lines: (i) MCF-7 cells as a well-established ER^+^ breast cancer cell line, since BCA2 has been reported to be upregulated in estrogen receptor positive (ER^+^) breast cancers (Burger et al., 2005, 2010; Kona et al., 2010), (ii) MDA-MB-231 as a prototype ER^–^ breast cancer cell line, (iii) MCF-12F as a cell line representing non-tumor breast cells, and (iv) HEK293T cells (transformed kidney cells) as an unrelated cell line and positive control, since previous findings from our group on certain characteristics of BCA2 were identified using this cell line (Nityanandam and Serra-Moreno, 2014; Colomer-Lluch and Serra-Moreno, 2017). Cells were transfected with plasmids coding for HA-BCA2 and a catalytically defective BCA2 mutant harboring alanine substitutions at cysteine residues that are critical for the functionality of the catalytic RING-finger domain (HA-C_228_-C_231_; Miyakawa et al., 2009; Nityanandam and Serra-Moreno, 2014; Colomer-Lluch and Serra-Moreno, 2017). As an additional negative control, cells were transfected with an empty vector. Parental cells were also included in these assays to assess their basal metabolic activity, as well as an additional control in which cells were serum-deprived (starvation treatment). The effect of overexpressing HA-BCA2 in cellular metabolism was measured for a total of 6 days after transfection. We found that HEK293T cells overexpressing HA-BCA2 exhibit reduced metabolic activity compared to cells transfected with the vector control or the catalytically defective BCA2 mutant (Figure 1A). The expression of HA-BCA2 and HA-C_228_-C_231_ was confirmed by western blot (Figure 1A; right panel), showing relatively similar levels of expression for both BCA2 constructs. These findings were corroborated under conditions of depleting BCA2. For this, stable cell lines constitutively expressing shRNAs targeting BCA2 were generated. Depletion of BCA2 was confirmed by transcriptional expression through reverse transcription followed by quantitative PCR (RT-qPCR; Figures 1B,D,F,H; right graphs), since the commercial antibodies against BCA2 cross-react with other cellular proteins, displaying multiple bands by western blotting that sometimes are difficult to discriminate from the BCA2 band. Metabolic activity was examined in the stably BCA2 knocked down cells in an analogous manner as in the overexpression studies, where day 0 represents measurement at 2 h after cells were plated. Consistent with the overexpression studies, downregulation of BCA2 in HEK293T cells caused an increase in metabolic activity compared to the shScrambled-treated cells, particularly at days 3 and 4, indicating that in this non-breast cell line BCA2 inherently reduces metabolic function. Unlike HEK293T cells, no significant differences were found between overexpression of wild type HA-BCA2, its catalytic-defective mutant or the vector control in ER^+^ cells (MCF-7) (Figure 1C), and this was further confirmed in the depletion assays. Although a decrease in metabolic activity was observed at days 3 and 4 of the time-course, by day 5 the metabolic activity for both the scrambled shRNA and the BCA2-depleted cells leveled out (Figure 1D). Of note, the kinetics for the parental and starved cells are the same for the overexpression and depletion graphs, since these experiments were performed in parallel for all the replicates, and this also applies to the other breast cell lines. With the exception of readings on day 3 post-transfection, overexpression of BCA2 had no major impact on metabolic activity in the ER^–^ cells (MDA-MB-231). Consistent with this, no major differences were found either under conditions of depleting BCA2 (Figure 1F). In non-tumor MCF-12F breast cells, up-regulation of BCA2 did not increase metabolic activity. However, its knockdown did, especially at day 4 (Figures 1G,H). Overall, these results indicate that BCA2 overexpression does not accelerate cellular metabolic activity.

### BCA2 Decreases Cell Proliferation and Migration in Non-tumor Epithelial Breast Cells

**FIGURE 2:**
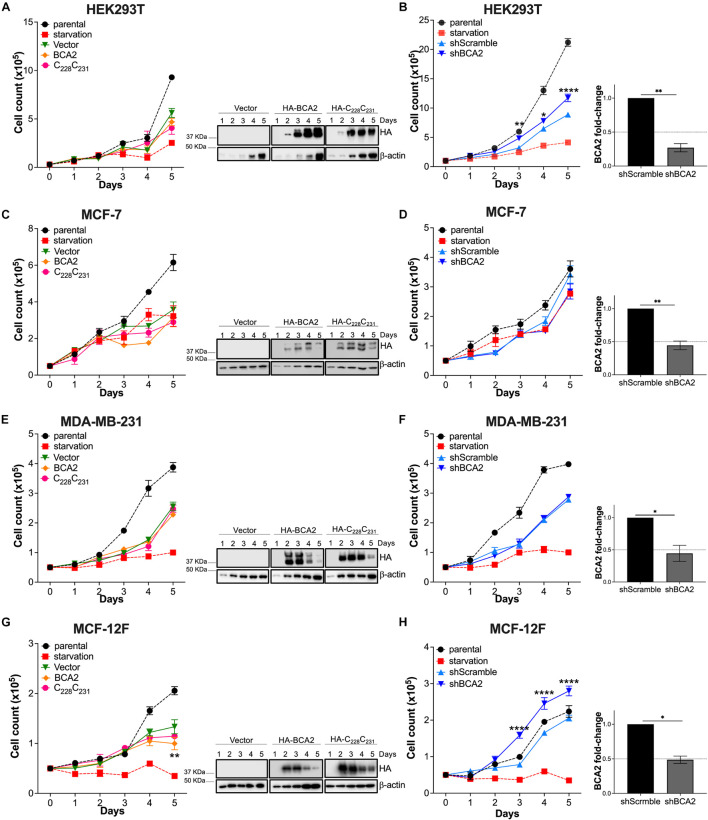
Impact of BCA2 levels on cell proliferation. **(A,C,E,G)** Cells, including HEK293T cells **(A)**, MCF-7 **(C)**, MDA-MB-231 **(E)**, and MCF-12F **(G)** were transfected with constructs encoding for HA-BCA2, a BCA2 catalytic-defective mutant (HA-C_22__8_C_231_) or an empty vector control. Cell proliferation was assessed by cell counting over a period of 6 days. As controls, parental cells and cells under nutrient deprivation were included. Representative western blots for the expression of HA-BCA2 and HA-C_22__8_C_231_ are shown next to the graphs. **(B,D,F,H)** Similar experiments were performed in cells stably transduced with a scrambled shRNA or depleted of BCA2. BCA2 depletion was verified on the first and last day of analysis by RT-qPCR (right panels show BCA2 levels on day 5 of the assay). Dotted lines represent threshold for biological significance. Data correspond to the mean and SEM of 3 independent experiments. *p < 0.05, **p < 0.01, ****p < 0.0001.

The XTT assay is a rapid method to assess overall metabolic function but does not identify what cellular events may be impacted by BCA2. For instance, BCA2 might be simultaneously intersecting with several cellular processes resulting in no net change in metabolic activity. Hence, we sought to investigate the effect of BCA2 on other cellular processes, like cell proliferation. Similar to the XTT assays, cells were transfected with HA-BCA2, HA-C_228_-C_231_ and the empty vector control, and cell proliferation was monitored for 6 days by cell counting. Of note, the transfection protocol caused some toxicity, since a delay in growth was observed in cells treated with the transfection reagent, even with an empty vector. Despite this effect, the impact of BCA2 on cell proliferation was reliably assessed by comparing growth kinetics with the empty vector and HA-C_228_-C_231_ transfected cells. Although HA-BCA2 significantly reduces cell metabolism in HEK293T cells, no significant differences in cell proliferation under conditions of overexpression were found (Figure 2A). HA-BCA2 and HA-C_228_-C_231_ expression levels were similar over the 6-day time period (Figure 2A; right panel). However, when BCA2 was depleted, an increase in cell proliferation was detected compared to the cells treated with the shscrambled RNA, and these differences were statistically significant (Figure 2B; right graph indicates the levels of depletion achieved). In the case of the ER^+^ MCF-7 cells, overexpression of HA-BCA2 caused no significant effects on cell growth. Similarly, no differences were detected when BCA2 was knocked down (Figures 2C,D). Overexpression of BCA2 caused no effects either on the MDA-MB-231 cells, and this was also verified by depleting endogenous BCA2 (Figures 2E,F). In the case of the non-tumor breast cells, overexpression of BCA2 caused only a marginal defect, although statistically significant, in cell growth at day 5. However, the depletion of this protein significantly accelerated cell proliferation, and these findings are consistent with the metabolic activity assays (Figure 1H), suggesting that BCA2 might arrest cell growth in non-tumor cells like MCF-12F.

**FIGURE 3:**
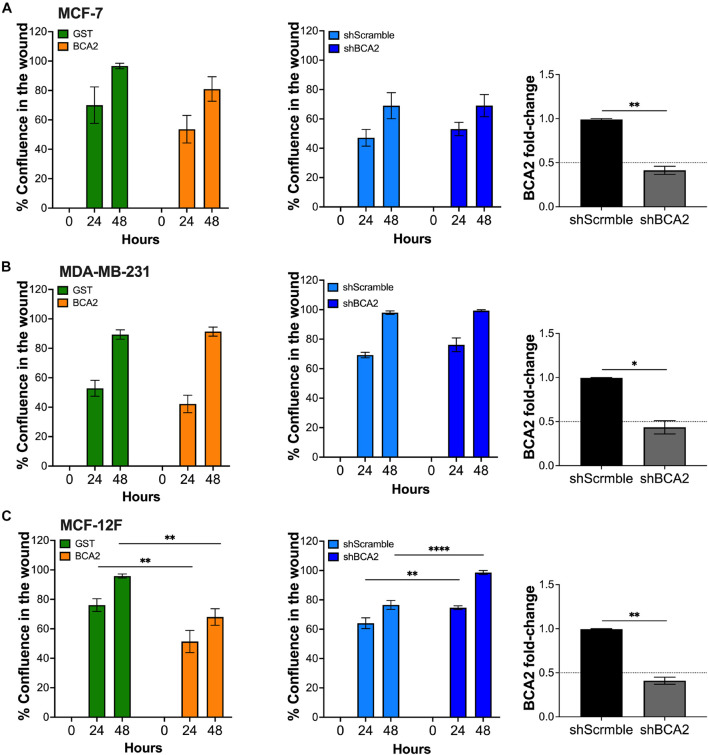
BCA2 reduces cell migration in non-tumor epithelial breast cells. Cells, including MCF-7 cells **(A)**, MDA-MB-231 **(B)** and MCF-12F **(C)** were transfected with constructs encoding for HA-BCA2 or HA-GST as an irrelevant protein control. When cells reached confluence, a scratch was performed on the monolayer. Cell migration and confluence was measured by live cell imaging for 48 h. Similar assays were performed in cells stably depleted of BCA2 or carrying a scrambled shRNA (middle panels). BCA2 knockdown was verified on the day the cells were plated by RT-qPCR (right panels). Dotted lines represent threshold for biological significance. Data correspond to the mean and SEM of three independent experiments. *p < 0.05, **p < 0.01, ****p < 0.0001.

Since MCF-12F cells grow at a lower rate than the breast cancer cell lines, we reasoned that the absence of effects on proliferation in the MCF-7 and MDA-MB-231 cells may be due to the fact that they reach confluence much faster. To investigate this possible scenario, we performed a scratch wound assay in the breast epithelial cells used in this study. For these assays, we transfected cells with HA-BCA2 or HA-GST. We used HA-tagged GST as an irrelevant protein and negative control rather than the empty vector control or the catalytic defective BCA2 mutant, since data in Figures 1, 2 showed that this motif is dispensable for BCA2’s effects on metabolic activity and cell proliferation in the breast cells. By including HA-GST, we were better positioned to rule out any possible effects on proliferation and migration due to protein overexpression that are not specific to BCA2’s actions. Besides overexpressing BCA2, additional migration assays were performed under conditions of BCA2 depletion. We found that up-regulation or depletion of BCA2 had no effect on cell migration in the cancer cell lines (Figures 3A,B and Supplementary Figures 1, 2). However, overexpression of BCA2 significantly reduced migration in the MCF-12F cells and this was corroborated in the depletion assays (Figure 3C and Supplementary Figures 1, 2). Hence, BCA2 inherently reduces cell proliferation and migration in the non-tumor epithelial breast MCF-12F cells.

### BCA2 Increases G1-to-S but Not S-to-G2/M Transition of the Cell Cycle

**FIGURE 4:**
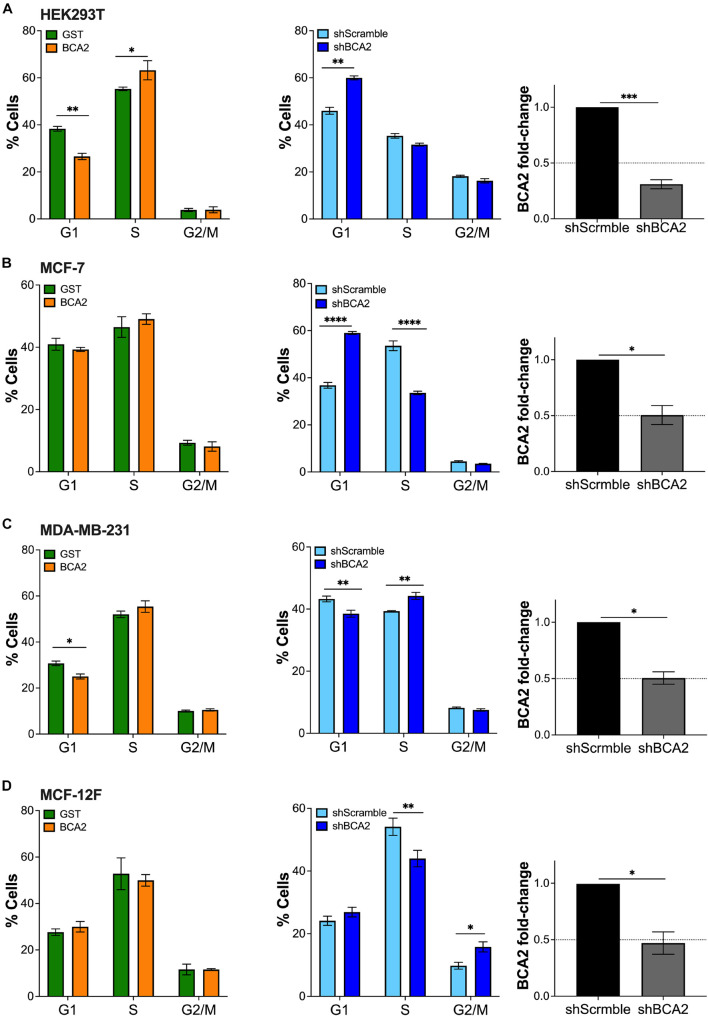
Up-regulation of BCA2 increases the transition from G1 to S phase. Cells, including HEK293T cells **(A)**, MCF-7 **(B)**, MDA-MB-231 **(C)**, and MCF-12F **(D)** were transfected with constructs encoding for HA-BCA2 or HA-GST as an irrelevant protein control. The abundance of cells in each phase of cell cycle was measured at the peak of HA-BCA2 expression (48 h post-transfection) by flow cytometry, after gating on the HA^+^ population (left panels). Similar assays were performed in cells stably depleted of BCA2 or carrying a scrambled shRNA (middle panels). BCA2 knockdown was verified on the day of analysis by RT-qPCR (right panels). Dotted lines represent threshold for biological significance. Data correspond to the mean and SEM of three independent experiments. *p < 0.05, **p < 0.01, ***p < 0.001, ****p < 0.0001.

Since previous studies reported that BCA2 promotes the degradation of p21 and c-Myc, which are important factors regulating cell cycle (Narita et al., 2012; Wang et al., 2013), we next investigated the effect of BCA2 on cell cycle progression. For this, we analyzed the number of cells present in each phase of the cell cycle under conditions of overexpression and depletion of BCA2 by flow cytometric assays using 7-AAD, a fluorescent dye that undergoes a spectral shift upon association with DNA. Hence, cell populations at different stages of the cell cycle can be discriminated depending upon their DNA content (Vignon et al., 2013). In the overexpression assays, cells were first gated on the HA^+^ population and subsequently separated among the different phases of the cell cycle depending on their 7-AAD content. In case of the depletion assays, the whole population was analyzed for cell cycle dynamics. For this, knockdown of BCA2 was verified by RT-qPCR on the same day of analysis (Figure 4; right panels). HEK293T cells overexpressing HA-BCA2 showed a significant decrease in G1 phase and a concomitant increase in S phase (Figure 4A; left panel), and this was corroborated by depleting BCA2, where an accumulation in G1 was observed (Figure 4A; middle and right panels).

Whereas no evident changes in cell cycle were detected in MCF-7 cells (ER^+^) overexpressing BCA2 (Figure 4B), the depletion of this protein led to a remarkable increase in G1 with a parallel reduction in S phase (Figure 4B; middle and right panels), suggesting that, at endogenous levels, BCA2 already exerts an effect on cell cycle and that overexpressing BCA2 beyond these levels has no additive effects. Similar to HEK293T cells, the up-regulation of BCA2 in the ER^–^ MDA-MB-231 reduced the number of cells in G1, although this decrease caused no effect on the subsequent cell cycle phases. Contrary to MCF-7 and HEK293T cells, depletion of BCA2 did not cause the expected increase in G1 and subsequent reduction in S phases in MDA-MB-231 cells (Figure 4C). On the contrary, a statistically significant reduction in G1 phase and an increase in S phase were observed.

Remarkably, up-regulation of BCA2 in the non-tumor MCF-12F cells did not affect cell cycle dynamics. However, depletion of endogenous BCA2 caused a shift in cell cycle distribution. Specifically, a significant reduction in S phase with a parallel increase in G1 and G2/M (Figure 4D). Whereas the redistribution to G1 phase was marginal, the shift to G2/M phase was statistically significant. Hence, similar to the MCF-7 cells, the endogenous levels of BCA2 in this non-tumor setting already exercise an effect on cell cycle entry and progression, so the up-regulation of BCA2 does not cause any additional effects. Furthermore, these findings suggest that BCA2 facilitates G1-to-S transition and a potential block on the transition to G2/M in this cellular context.

### BCA2 Maintains Its Ability to Modulate NF-κB, but Exerts Opposite Effects on IRF1 Regulation in ER^–^ Tumor and Non-tumor Breast Cell Lines

**FIGURE 5:**
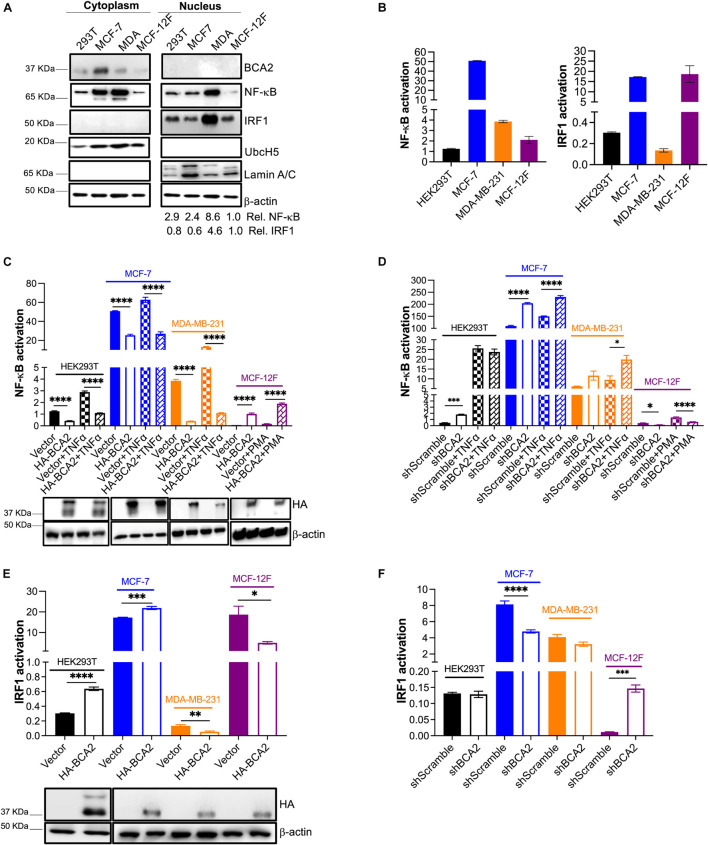
BCA2 preserves its ability to regulate NF-κB, but displays distinct roles in the regulation of IRF1 depending on the cellular context. **(A,B)** The endogenous levels of NF-κB and IRF1 activation for HEK293T cells, MCF-7, MDA-MB-231, and MCF-12F were investigated by subcellular fractionation **(A)** and using luciferase reporter assays for each of these transcription factors **(B)**. **(C)** The effects of overexpressing HA-BCA2 on NF-κB activation were measured by luciferase reporter assays on HEK293T cells, MCF-7, MDA-MB-231, and MCF-12F. Expression of HA-BCA2 was verified by western blot (bottom panels). **(D)** Complementary assays were performed by depleting the endogenous levels of BCA2. BCA2 depletion was verified on the day of analysis by RT-qPCR (not shown). **(E)** The effects of overexpressing HA-BCA2 on IRF1 activation were measured by luciferase reporter assays on HEK293T, MCF-7, MDA-MB-231, and MCF-12F cells. Expression of HA-BCA2 was verified by western blot (bottom panels). **(F)** Complementary assays were performed by depleting the endogenous levels of BCA2. BCA2 depletion was verified on the day of analysis by RT-qPCR (not shown). *p < 0.05, **p < 0.01, ***p < 0.001, ****p < 0.0001. Data correspond to the mean and SEM of 3 independent experiments.

Although BCA2 has been reported to modulate oncogenic and tumor suppressor molecules (Narita et al., 2012; Smith et al., 2013; Wang et al., 2013), our findings so far indicate that, despite an effect on G1-to-S transition, BCA2 does not accelerate metabolic activity, cell proliferation nor migration. To investigate if the connection between BCA2 levels and transformation is due to an impairment in BCA2’s enzymatic activities, we assessed BCA2’s effect on the regulation of NF-κB. Our lab previously demonstrated that BCA2 is induced by NF-κB and that it provides a negative feedback loop through the SUMOylation of IκBα. NF-κB not only is a critical transcription factor for HIV RNA synthesis, but also a factor that facilitates cell proliferation in many cancers (Kretzschmar et al., 1992; West et al., 2001; Biswas et al., 2004; Hayden et al., 2006; Xia et al., 2014; Blakely et al., 2015). Hence, we decided to investigate the effects of ectopically expressed BCA2, as well as endogenous BCA2, on NF-κB in different breast epithelial cellular contexts. In addition to NF-κB, we also found that BCA2 activates IRF1, a well-known immunomodulatory transcription factor and tumor suppressor (Bouker et al., 2005; Colomer-Lluch and Serra-Moreno, 2017). Although this activity of BCA2 is independent of its enzymatic activities (Colomer-Lluch and Serra-Moreno, 2017), we sought to explore if this role of BCA2 is preserved in breast cancers and non-tumor cells. For this, the basal activation of NF-κB and IRF1 were measured by subcellular fractionation and findings were confirmed using luciferase-reporter assays. In their inactive status, NF-κB and IRF1 are found sequestered in the cytosol (Oeckinghaus and Ghosh, 2009; Murtas et al., 2013). Therefore, detection of these transcription factors in the nucleus would suggest that they are active. Compared to the non-tumor breast cells, the nuclear levels of NF-κB were higher in the transformed and breast cancer cell lines (Figure 5A). With the exception of the MDA-MB-231 ER^–^ cells, the transformed cells displayed similar nuclear levels of IRF1 to the non-tumor breast cells. Endogenous BCA2 was only detected in the cytosol and not in the nucleus of the cells we tested (Figure 5A). The purity of the fractions was confirmed using the nuclear marker Lamin A/C and the cytosolic marker UbcH5, as previously reported (Colomer-Lluch and Serra-Moreno, 2017). To confirm the activation status of NF-κB and IRF1, luciferase reporter assays were performed in HEK293T, MCF-7, MDA-MB-231, and MCF-12F cells. Cells were co-transfected with a vector coding for the luciferase gene under the control of a NF-κB- or IRF1-inducible promoter. Compared to the non-tumor MCF-12F cells, ER^+^ MCF-7 cells displayed extremely high basal NF-κB activation levels, although by looking at the fractionation assays, the amount of NF-κB in their nucleus did not seem as high. Consistent with their carcinogenic nature, ER^–^ MDA-MB-231 cells also had higher levels of NF-κB activation than the MCF-12F cells, although they did not reach the levels observed in the MCF-7 cells. By contrast, HEK293T cells had similar NF-κB activation levels as the MCF-12F cells (Figure 5B; left panel). Regarding IRF1 activation, HEK293T and MDA-MB-231 cells showed considerably lower IRF1 activation than the non-tumor breast cells. Remarkably, once again MCF-7 cells had the highest basal activation levels of IRF1 (Figure 5B; right panel). These results indicate that the nuclear levels of these proteins are not an accurate reflection of their activation status. In consequence, luciferase reporter assays were used to reliably measure NF-κB and IRF1 activation for the rest of this study. After uncovering the basal activation levels of these transcription factors, we next assessed the effect of overexpressing and depleting BCA2 in these cells on NF-κB and IRF1. For this, all cells were co-transfected with an empty vector control or HA-BCA2 along with the luciferase reporter gene and β-galactosidase plasmid. Thirty-six hours later, cells were incubated with DMSO, TNFα or PMA to activate NF-κB. Of note, PMA was only used as an alternative to TNFα in MCF-12F cells, since these cells did not respond to TNFα (data no shown). Forty-eight hours post-transfection, NF-κB activation was measured by luminescence, as explained above. The level of BCA2 expression was examined by western blotting (Figure 5C; bottom). As we previously reported, NF-κB activation was down-regulated by HA-BCA2 in HEK293T and also in ER^+^ MCF-7 and ER^–^ MDA-MB-231 cells, even when we triggered NF-κB by TNFα stimulation. However, the opposite effect was observed in the non-tumor MCF-12F cells (Figure 5C). As indicated earlier, MCF-12F cells were not responsive to TNFα treatment to trigger canonical NF-κB, which suggests that their ability to respond to NF-κB-inducing stimuli is limited. In order to examine the role of endogenous BCA2 in NF-κB activation, similar assays were performed in cells stably depleted of BCA2 and/or expressing an irrelevant shRNA (shScramble). Confirmation of depletion was always performed before any assay by RT-qPCR (not shown). Consistent with the overexpression studies, depletion of BCA2 caused an increase in the basal activation levels of NF-κB for HEK293T, MCF-7, and MDA-MB-231 cells. Likewise, consistent with the effect of overexpressing BCA2 in non-tumor MCF-12F cells, depletion of this protein reduced the basal activation levels of NF-κB (Figure 5D).

We next assessed the role of BCA2 in IRF1 activation. Whereas IRF1 activation was increased in HEK293T and ER^+^ MCF-7 cells overexpressing HA-BCA2, its activation was decreased in ER^–^ MDA-MB-231 and non-tumor MCF-12F cells (Figure 5E). Accordingly, no effect or a reduction in IRF1 activation was detected in BCA2-knocked down HEK293T cells, MCF-7, and MDA-MB-231 cells. However, the activation of IRF1 was increased in non-tumor breast cells (MCF-12F) depleted of BCA2 (Figure 5F). These results indicate that BCA2 exerts cell-type-specific effects on the regulation of IRF1. In sum, BCA2 is able to block NF-κB and activate IRF1 in ER^+^ breast cancer cell lines, but exhibits opposite effects on these molecules in other cell types.

### BCA2 Uses Its Zinc-Finger (BZF) Domain to Interact With IRF1 and Facilitate Its Nuclear Translocation

**FIGURE 6:**
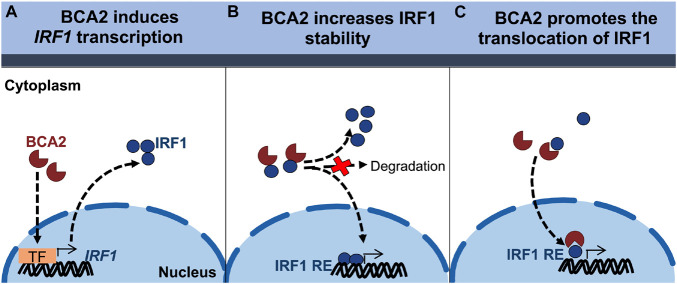
Proposed models for the BCA2-mediated activation of IRF1. **(A)** BCA2 increases IRF1 activity by upregulating its expression. **(B)** BCA2 increases IRF1 activity by increasing its stability. **(C)** BCA2 increases IRF1 activity by facilitating its nuclear transport. *TF, transcription factor; RE, responsive element*.

**FIGURE 7:**
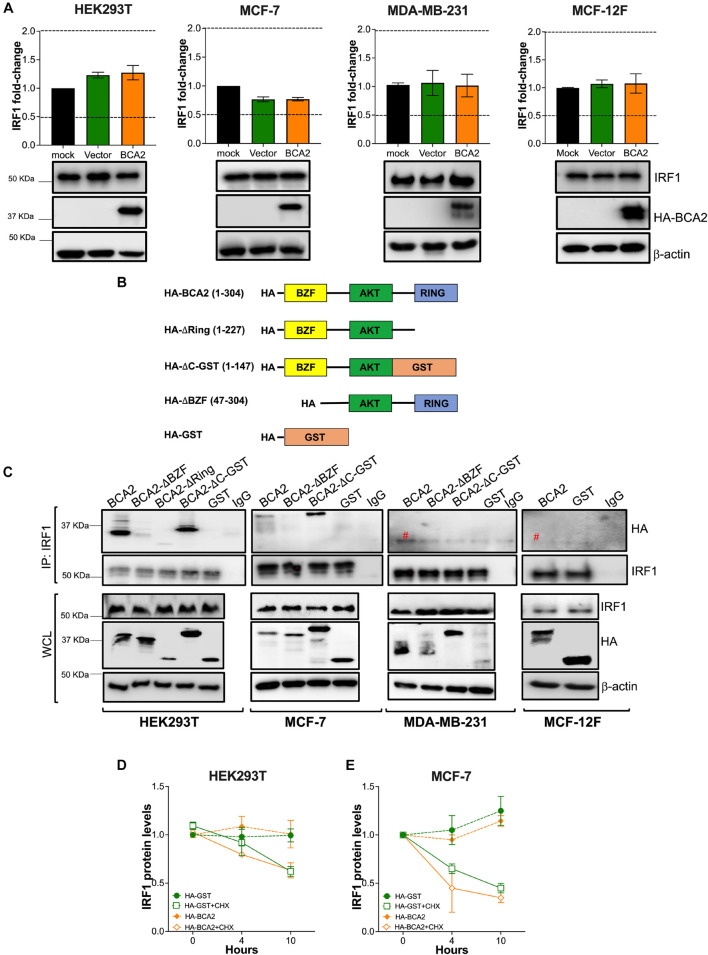
BCA2 uses its Zinc-finger domain to interact with IRF1 in HEK293T and MCF-7 cells. The hypotheses formulated in Figure 6 were tested in HEK293T, MCF-7, MDA-MB-231, and MCF-12F cells. **(A)** The effects of up-regulating BCA2 on IRF1 gene expression was assessed by RT-qPCR and western blot. Dotted lines represent threshold for biological significance. Data correspond to the mean and SEM of 3 independent experiments. Up-regulation of BCA2 and endogenous IRF1 levels were verified by western blot (bottom panels). **(B)** Diagram of the BCA2 truncation mutants that were generated to test models (on Figures 6B,C). The numbers in brackets indicate the BCA2 residues present in each mutant. **(C)** BCA2-IRF1 binding was investigated in all the cell lines by immunoprecipitating endogenous IRF1 and assessing the presence of the HA-tagged constructs presented in panel B in the pulldown fraction. The levels of expression of IRF1, the BCA2 truncation constructs and β-actin were examined from the whole cell lysates (WCL). Red pound symbol indicates bands corresponding to the light chain of the antibody used in the IP. Blots are representative of 3 independent experiments. **(D,E)** The effect of HA-BCA2 and HA-GST (irrelevant protein) on IRF1 protein stability was investigated in HEK293T and MCF-7 cells in the presence and absence of cycloheximide (CHX), a translation inhibitor. Samples were collected over the course of 10 h after DMSO or CHX treatment. The expression or IRF1 over β-actin and relative to the zero time-point was calculated by densitometry analyses and plotted as IRF1 relative levels. Data correspond to the mean and SEM of 3 independent experiments.

Although the effect of BCA2 on NF-κB in non-tumor breast cells was unexpected, the contrasting effects that BCA2 exerts on IRF1 made us hypothesize that this distinct regulation might help explain the current controversies on the role of BCA2 in cancer development. In order to uncover the mechanism by which BCA2 activates IRF1, we evaluated the following possible scenarios: (a) BCA2 activates IRF1 by increasing *IRF1* transcription; (b) BCA2 activates IRF1 through protein-protein interactions, by increasing IRF1 stability and consequently its activity; and (c) BCA2 facilitates IRF1 transport to the nucleus, since BCA2 has the ability to shuttle between the cytosol and the nucleus (Amemiya et al., 2008; Colomer-Lluch and Serra-Moreno, 2017; Figure 6). As a first approach to investigate this, we assessed the transcriptional levels of *IRF1* upon BCA2 overexpression by RT-qPCR. HEK293T, MCF-7, MDA-MB-231, and MCF-12F cells were transfected with only the transfection reagent (mock), an empty vector or HA-BCA2. Forty-eight hours post-transfection, the transcriptional levels of *IRF1* were measured by RT-qPCR. The fold-change in *IRF1* expression was normalized to *GAPDH* and expressed as relative levels compared to the mock control. HA-BCA2 and IRF1 levels were confirmed by western blot (Figure 7A; bottom). No significant differences in *IRF1* expression were observed between vector and HA-BCA2 in HEK293T, MCF-7, MDA-MB-231, and MCF-12F cells, indicating that the BCA2-dependent modulation of IRF1 in these cells is not due to gene induction or down-regulation (Figure 7A). We next examined the protein-protein interactions between BCA2 and IRF1. For this, HEK293T, MCF-7, MDA-MB-231, and MCF-12F cells were transfected with HA-BCA2 or different truncation mutants of BCA2 such as a BCA2 mutant where the RING domain is deleted (HA-BCA2-△Ring), a mutant where the whole C-terminal region is deleted (HA-BCA2-△C-GST), and a mutant lacking the zing-finger domain (HA-ΔBZF). Of note, GST was introduced to replace the C-terminus of BCA2 in the HA-BCA2-△C-GST mutant because the deletion of the C-terminal region of BCA2 significantly reduced the stability of the truncated protein. In consequence, HA-GST was included as an irrelevant protein control (Figure 7B). The ability of BCA2 and BCA2 mutants to interact with IRF1 was determined by co-immunoprecipitation (co-IP) where endogenous IRF1 was selectively immunoprecipitated. Of note, due to the difficulty of propagating MCF-12F cells for these co-IPs, only IRF1-BCA2 and IRF1-GST interactions were examined in these cells. Whereas no association between BCA2 and IRF1 was observed in MDA-MB-231 and MCF-12F cells, BCA2, BCA2-△Ring, BCA2-△C-GST but not BCA2-ΔBZF or GST were detected in the IRF1 pulled down fraction of HEK293T and MCF-7 cells (Figure 7C), indicating that BCA2 physically interacts with IRF1 most likely through its N-terminal Zinc-finger region. Although the interaction between IRF1 and BCA2-△Ring was significantly diminished (Figure 7C; left blot, 3rd lane), this mutant has considerably lower expression levels than the other mutants tested, which would explain the low amount of this protein in the pulldown fraction. In addition, the fact that the BCA2-△C-GST mutant still immunoprecipitated with IRF1 further supports this hypothesis. Both BCA2-△Ring and BCA2-△C-GST are very similar (there is an extra 80 amino acid truncation in the BCA2-△C-GST mutant; see Figure 7B), yet the expression of BCA2-△C-GST is comparable to that of HA-BCA2 and this mutant clearly interacts with IRF1, indicating that the determinants for the interaction between BCA2 and IRF1 are present in this mutant and, by analogy, they should be present in the BCA2-△Ring mutant. Due to the low levels of expression of the BCA2-△Ring truncation mutant, we decided to not include this construct in the co-IPs for the other cell lines.

The fact that BCA2 physically interacts with IRF1 in HEK293T and MCF-7 cells opens the possibility of hypotheses (b) and (c) depicted in Figure 6. First, we assessed if this association increases the stability of IRF1 in HEK293T and MCF-7 cells, since in these cell lines BCA2 up-regulates IRF1 activity. For this, IRF1 expression levels were compared between cells treated with DMSO or Cycloheximide (CHX), a compound that blocks translation. No differences were observed in the protein levels of IRF1 between HA-GST and HA-BCA2 transfected cells, even when translation was inhibited (Figures 7D,E and Supplementary Figure 3). Hence, these results indicate that BCA2 does not increase IRF1 stability.

**FIGURE 8:**
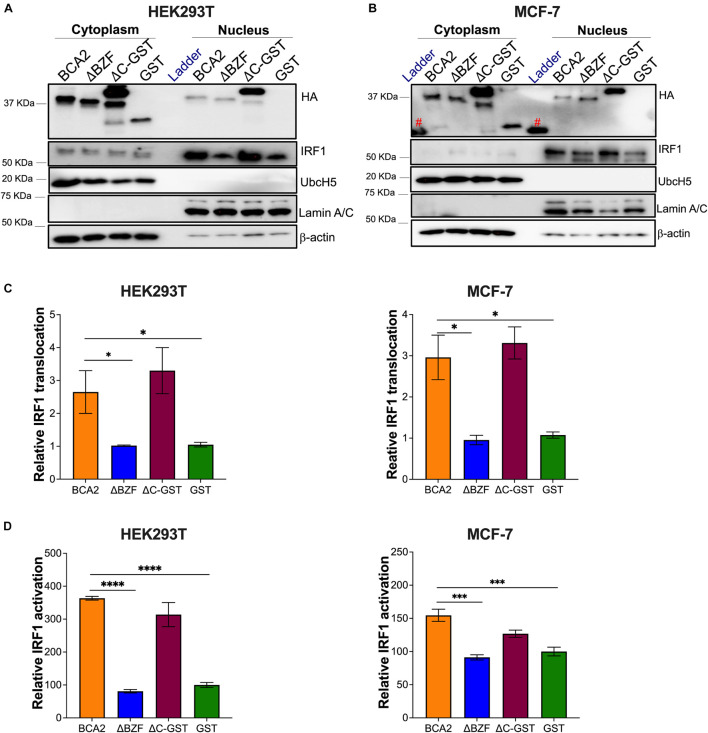
BCA2 promotes the nuclear translocation and activation of IRF1 in HEK293T and MCF-7 cells. **(A,B)** Subcellular fractionation assays in HEK293T and MCF-7 cells were performed to determine the levels of cytosolic vs. nuclear IRF1 in the presence of HA-BCA2 and its truncation mutants. The blots are representative of 3 independent experiments. **(C)** The IRF1 nuclear translocation relative to GST was quantified by densitometry analyses by normalizing nuclear IRF1 to Lamin A/C. **(D)** Data was verified using IRF1 luciferase reporter assays. *p < 0.05, ***p < 0.001, ****p < 0.0001. Data correspond to the mean and SEM of 3 independent experiments. *IP, immunoprecipitation; Red pound symbol, unspecific bands due to cross-reactivity of the antibody with the protein ladder.*

We next assessed whether BCA2 increases the nuclear translocation of IRF1 in these cells. For this, HEK293T and MCF-7 cells were transfected with wild-type BCA2, BCA2-ΔBZF, BCA2-△C-GST, or GST. Cytosolic and nuclear fractions were extracted as described earlier and the levels of IRF1 in the nucleus were assessed. Remarkably, BCA2 increased the levels of nuclear IRF1 by > 2-fold. However, nuclear IRF1 levels remained unchanged in the presence of GST or the BCA2-ΔBZF mutant, suggesting that the BCA2-IRF1 interaction facilitates IRF1 transport to the nucleus, which consequently increases its ability to interact with its responsive genes (Figures 8A–C). This hypothesis was further verified by IRF1 luciferase reporter assays. While overexpression of BCA2 caused an increase in IRF1 activity, the BCA2-ΔBZF mutant did not affect the basal levels of IRF1 activity (Figure 8D). Hence, these findings confirm that, in HEK293T and MCF-7 cells, BCA2 activates IRF1-mediated responses by facilitating the nuclear translocation of this transcription factor.

## Discussion

Breast cancer-associated gene 2 (BCA2) is an E3 ubiquitin and SUMO ligase that restricts retroviral replication, including HIV, by (i) down-regulating the transcription factor NF-κB (this is achieved through the SUMOylation of IkBα) (Colomer-Lluch and Serra-Moreno, 2017), (ii) promoting the ubiquitination and lysosomal degradation of the HIV protein Gag (Nityanandam and Serra-Moreno, 2014), and (iii) enhancing the antiviral activity of Tetherin/BST2 (Miyakawa et al., 2009). Before its antiviral actions were recognized, BCA2 was a well-known marker for breast cancer, particularly ER^+^ cancers, since its up-regulation correlated with ER status and poor prognosis (Burger et al., 2005; Burger et al., 2006). However, whether BCA2 overexpression is a contributing factor to cancer development was poorly understood. Hence, understanding the role of BCA2 in cancer development is crucial to gauge BCA2’s candidacy for antiretroviral therapy. Several studies have tried to address this with mixed results. Whereas some reports point to an oncogenic role for BCA2, others claim the opposite. These contrasting findings may be the result of investigating interactions between BCA2 and specific cellular targets and/or using a particular cell line. In an effort to bring consensus, we investigated the role of BCA2 in cellular metabolic activity, cell proliferation, cell cycle progression, and cell migration in different breast cellular environments. In addition, we assessed whether the SUMO ligase activity of BCA2 was preserved in different cellular contexts as well as its ability to activate the IRF1 tumor suppressor.

A potential role for BCA2 in altering cellular metabolic activity was investigated in ER^+^ (MCF-7), ER^–^ (MDA-MB-231), non-tumor breast cells (MCF-12F) as well as in HEK293T cells. We included HEK293T cells because many of our observations on the role of BCA2 as an E3 ubiquitin and SUMO ligase were obtained in this cell line (Nityanandam and Serra-Moreno, 2014; Colomer-Lluch and Serra-Moreno, 2017). Our survey showed that BCA2 reduces cellular metabolic activity in HEK293T and modestly in non-tumor breast cells but did not affect the overall metabolism of MCF-7 and MDA-MB-231 cells. Since the metabolic activity assays only give us a partial picture of the potential effects of BCA2 on tumor development and cancer progression, we next assessed whether BCA2 affects cell proliferation and migration and found that, with the exception of the non-tumor MCF-12F cells, in which endogenous BCA2 seems to reduce cell proliferation and migration, BCA2 has no major impact on these processes in the breast cancer cell lines analyzed here. We next examined the effects of BCA2 on cell cycle dynamics. Overexpression of BCA2 increased progression from G1 to S phase, particularly in HEK293T cells. Accordingly, upon BCA2 knockdown, a redistribution to G1 phase was observed for HEK293T, MCF-7 and slightly in MCF-12F cells but not for the MDA-MB-231 cells. Of note, besides the marginal increase in G1, redistribution to G2/M was also detected in the BCA2-depleted non-tumor cells. The fact that the overexpression of BCA2 did not cause much of an effect in cell cycle distribution in MCF-7 cells is in accordance with the high BCA2 expression levels that these cells inherently display (Figure 5A), so up-regulation beyond these levels has little to no impact on G1-to-S transition. In agreement with this hypothesis, the depletion of BCA2 in MCF-7 cells caused a massive accumulation in G1 with a parallel decrease in S phases. A similar phenomenon was observed in the MCF-12F cells, even though these cells have the lowest level of expression of BCA2 among our cell lines (Figure 5A). Hence, we conclude that the endogenous levels of BCA2 in MCF-7 and MCF-12F cells are sufficient to alter cell cycle dynamics. Similar to the HEK293T cells, overexpression of BCA2 caused a decrease in G1 phase in the ER^–^ cells (MDA-MB-231). By contrast, the depletion of BCA2 led to a significant reduction in G1 and an increase in S phases. These findings suggest that the endogenous levels of BCA2 in these ER^–^ cells do not reach a specific (yet unknown) threshold for it to stimulate G1-to-S entry.

The effect of BCA2 on the release from G1 to S phase is consistent with previous reports showing that BCA2 promotes the degradation of the tumor suppressor p21 (Wang et al., 2013), which would otherwise cause an arrest in G1. Hence, despite its little impact on metabolic activity, BCA2 facilitates entry in S phase. However, if BCA2 acts by accelerating cell cycle progression, an increase in G2/M would have been expected under conditions of overexpression (Taira et al., 2012). Therefore, although BCA2 is sufficient to bypass the G1-to-S checkpoint, either its sole overexpression is not enough to bypass the S-to-G2 checkpoint, or it may play a restrictive role in the subsequent phases of cell cycle. Besides promoting the degradation of p21, BCA2 has also been reported to down-regulate c-Myc (Narita et al., 2012). c-Myc is known to modulate the expression of genes that promote proliferation, including genes that facilitate mitosis (M phase) (Bretones et al., 2015; Yang et al., 2018). Hence, the BCA2-mediated down-regulation of c-Myc may account for the absence of an enrichment in G2/M under conditions of overexpression. However, if this were the case a corresponding increase in G2/M would have been observed when BCA2 was depleted in the breast cancer and HEK293T cells. A redistribution to G2/M was in fact detected in the non-tumor MCF-12F breast cells. Hence, these observations indicate that the role of BCA2 at promoting G1-to-S transition is preserved for the most part across cell types, while its role at blocking entry to G2/M – likely through the down-regulation of c-Myc—is only maintained in the non-tumor cells. These findings are consistent with the transformation process that the other cells are undergoing, in which other cellular factors regulating the S-to-G2/M checkpoint may have been altered. Nevertheless, the fact that BCA2 promotes G1-to-S transition but not entry in the following phases of cell cycle is in line with our proliferation and migration assays, in which up-regulation of BCA2 did not significantly affect cell growth. If anything, endogenous BCA2 delays cell proliferation in the non-tumor MCF-12F cells.

So far, the data discussed above show no evident pro-oncogenic role for BCA2 in these cellular environments. Thus, we next investigated whether the connection between BCA2 and cancer development is due to a defect in its enzymatic properties. For this, we studied the SUMO E3 ligase function of BCA2 at regulating NF-κB. Our previous work revealed that besides being an E3 ubiquitin ligase, BCA2 also serves as an E3 SUMO ligase. Specifically, we found that BCA2 promotes the SUMOylation of IkBα, a post-translational modification that prevents targeting IkBα for proteasomal degradation. Hence, this activity of BCA2 makes IkBα more stable and a stronger inhibitor of NF-κB (Colomer-Lluch and Serra-Moreno, 2017). In addition to its well-known role in innate immunity, NF-κB also regulates genes involved in cell proliferation. In fact, NF-κB is often hyperactivated in cancer cells, and this was corroborated here, since, unlike the non-tumor breast cells, both ER^+^ and ER^–^ breast cancer cell lines exhibited high levels of NF-κB activation (Figure 5B). Despite these high levels of NF-κB, we found that the overexpression of BCA2 effectively down-regulated the activity of this transcription factor in all cell types investigated, except for the MCF-12F cell line. In agreement with these findings, the depletion of endogenous BCA2 caused the opposite effect, indicating that—with exception of the MCF-12F cells—even at its endogenous levels, BCA2 can down-regulate NF-κB. Unlike the other cells in this study, MCF-12F cells are insensitive to TNFα stimulation, regardless of what concentration we used to stimulate them (not shown). Since the TNFα receptor (TNFR) is ubiquitously expressed, we reasoned that these cells either lack this receptor or downstream effectors of the canonical NF-κB pathway. This is relevant, since the BCA2-mediated regulation of NF-κB is through the canonical pathway (Colomer-Lluch and Serra-Moreno, 2017). We then used PMA as an alternative trigger for NF-κB, since PMA activates both canonical and non-canonical NF-κB. Under these conditions we were able to trigger NF-κB signaling, albeit very modestly. However, not only BCA2 was unable to down-regulate NF-κB, but also induced its activation in these cells. This is in contrast with our previous and current findings in the HEK293T, MCF-7, and MDA-MB-231 cells. Since the MCF-12F cells already exhibited an unexpected phenotype in NF-κB signaling, we decided to use MCF-10A as an alternative model for non-tumor breast cells. Unfortunately, we found that these cells are extremely resistant to transfection. Even when we managed to overexpress HA-BCA2 and deplete the endogenous protein through retroviral transduction, we were unable to transfect these cells with the luciferase and β-galactosidase reporter plasmids, regardless of the transfection reagent or conditions. Hence, because of their unusual lack of response to TNFα, at this moment we are unclear if the effect of overexpressing BCA2 on NF-κB in MCF-12F cells is an accurate representation of the role of this E3 ubiquitin and SUMO ligase in the regulation of NF-κB in non-tumor breast tissue.

In addition to NF-κB, our previous work uncovered that BCA2 also regulates IRF1, a well-known tumor suppressor. Although this activity of BCA2 is independent of its catalytic activity, we investigated whether BCA2 loses control over IRF1 in the cancer environment. Whereas BCA2 activates IRF1 in HEK293T and MCF-7 (ER^+^) cells, it causes IRF1 down-regulation in ER^–^ and non-tumor breast cell lines. These contrasting findings may explain, at least in part, the contradictory observations reported for BCA2’s role in cancer formation. Hence, we decided to elucidate the mechanism by which BCA2 activates IRF1 in certain cell types, with the hope that we could understand why it exerts opposite roles in the other cells. Our first hypothesis was that BCA2 promotes the up-regulation of *IRF1*. RT-qPCR assays demonstrated no differences in the mRNA levels of IRF1 when overexpressing BCA2. Our second hypothesis was that BCA2 increases IRF1 stability through a physical interaction. In fact, we found that BCA2 uses its Zinc-finger (BZF) domain to associate with IRF1, but only in HEK293T and ER^+^ MCF-7 cells. However, this interaction has no effects on IRF1 stability. The third hypothesis was that BCA2 association with IRF1 in HEK293T and MCF-7 cells helps shuttle this transcription factor to the nucleus, which consequently causes an up-regulation of IRF1 responsive genes. Cellular fractionation assays, in which we used the ΔBZF mutant as a negative control, demonstrated that BCA2 significantly increases the nuclear translocation of IRF1, and its nuclear levels correlate with the up-regulation of IRF1 target genes. Besides its function as a transcription factor, IRF1 has been found to increase apoptotic activity as part of its tumor suppressive roles (Bouker et al., 2005). Although we did not directly evaluate this activity in the current study, our data on cell proliferation show that the endogenous levels of BCA2 delay cell growth in HEK293T cells, one of the cellular contexts where BCA2 promotes IRF1 activation. These observations would be consistent with a potential role for IRF1 in promoting apoptosis, although this hypothesis needs to be experimentally tested.

Contrary to the HEK293T and MCF-7 cells, BCA2 was unable to interact with IRF1 in the non-tumor and ER^–^ cancer cells, which is consistent with a lack of BCA2-dependent IRF1 up-regulation. Nevertheless, this lack of IRF1-BCA2 interaction does not explain the negative regulatory effect that BCA2 exerts on IRF1 in these cellular environments. qPCR assays showed no evidence that BCA2 decreases *IRF1* expression in these cells. Hence, the reduction in IRF1 activity must be caused by other effects of BCA2 on IRF1 or on cellular factors that modulate IRF1 functionality. In the case of non-tumor cells, cellular sensors for transformation switch IRF1 actions from basal to active (during transformation events) or to inactive (when the insults have already been resolved). Many of these switches involve post-translational modifications of IRF1, including SUMOylation (Park et al., 2007; Feng et al., 2021). Although BCA2’s enzymatic activity is dispensable to activate IRF1 in HEK293T and MCF-7 cells, and we were unable to detect a physical interaction between BCA2 and IRF1 in the non-tumor cell line, it is plausible that BCA2 contributes to such regulation to some extent. If so, this raises the possibility that BCA2 plays dual roles in the modulation of IRF1: able to increase IRF1 nuclear translocation under conditions of transformation, and at the same time capable of down-regulating IRF1 activity when pro-transforming events have been rectified. The fact that BCA2 fails at inducing the anti-tumor function of IRF1 in the MDA-MB-231 ER^–^ cell line is consistent with the aggressive nature of these tumor cells, which may have evolved mechanisms to override BCA2 actions. In fact, compared to the other cells used in this study, the MDA-MB-231 cells exhibited the highest nuclear levels of IRF1, suggesting that these cells may be trying to regain control over cell proliferation by up-regulating this tumor suppressor. Despite its up-regulation, IRF1 is either dysfunctional (as observed in our luciferase assays) or cannot override the transformation process in these cells, which is consistent with their aggressive growth. Besides uncovering if/how BCA2 plays a dual role in the regulation of IRF1 (in non-transforming vs. transforming settings), future work will aim at examining how BCA2 levels affect cell cycle progression, differentiation, and proliferation in HIV target cells. This information is crucial before we explore the feasibility of delivering ectopic BCA2 to HIV-infected cells as a therapeutic approach against this virus.

In summary, in this study we show that, despite promoting entry to S phase of the cell cycle, overexpression of BCA2 has no significant impact on cell proliferation in the different breast epithelial contexts tested here. However, the role of BCA2 in the regulation of IRF1 differs depending on the cell type. These distinct actions may account, at least in part, for the different proposed roles for BCA2 in breast cancer. Hence, elucidating the mechanisms of regulation of IRF1 by BCA2 in different cellular contexts will bring us closer to understanding the relationship between BCA2, cancer development and prognosis.

## Data Availability Statement

The original contributions presented in the study are included in the article/Supplementary Material, further inquiries can be directed to the corresponding author/s.

## Author Contributions

YS and RS-M designed the experiments and wrote the manuscript. YS performed the experiments and analyzed the data. SC-G and YC provided technical support. All authors contributed to the article and approved the submitted version.

## Conflict of Interest

The authors declare that the research was conducted in the absence of any commercial or financial relationships that could be construed as a potential conflict of interest.

## Publisher’s Note

All claims expressed in this article are solely those of the authors and do not necessarily represent those of their affiliated organizations, or those of the publisher, the editors and the reviewers. Any product that may be evaluated in this article, or claim that may be made by its manufacturer, is not guaranteed or endorsed by the publisher.

## Supplementary Material

Supplementary Figure 1 Overexpression of BCA2 does not accelerate cell migration. Cell migration was assessed in MCF-7, MDA-MB-231, and MCF-12F cells transfected with HA-GST or HA-BCA2 over the course of 48 h. When cells reached > 90% confluence, the monolayer was scratched, and cell migration was monitored by live cell imaging. Scale bar: 1,000 μm.

Supplementary Figure 2 Depletion of BCA2 delays cell migration in non-tumor breast epithelial cells. Cell migration was assessed in MCF-7, MDA-MB-231, and MCF-12F cells stably transduced with shScrambled RNA or shRNAs specific for BCA2. BCA2 knockdown was verified by RT-qPCR on the day the cells were plated (not shown). When cells reached > 90% confluence, the monolayer was scratched, and cell migration was monitored by live cell imaging. Scale bar: 1,000 μm.

Supplementary Figure 3 BCA2 has no effects on IRF1 protein stability. **(A,B)** HEK293T cells **(A)** and MCF-7 cells **(B)** were transfected with HA-BCA2 or HA-GST (irrelevant proteins). 48 h later, cells were treated with DMSO or 200 μM of CHX for 10 h. Cells were harvested at 0, 4, and 10 h after CHX treatment. The relative levels of IRF1 are displayed underneath the blots, after correcting with β-actin levels. Blots are representative of 3 independent experiments. *Red pound symbol: unspecific bands*.
