# Supplementary figures and images for: Effects of the SUMO Ligase BCA2 on Metabolic Activity, Cell Proliferation, Cell Migration, Cell Cycle, and the Regulation of NF-κB and IRF1 in Different Breast Epithelial Cellular Contexts
Source: Front Cell Dev Biol. 2021 Sep 13;9:711481. doi: 10.3389/fcell.2021.711481 (PMC8473798; doi:10.3389/fcell.2021.711481)

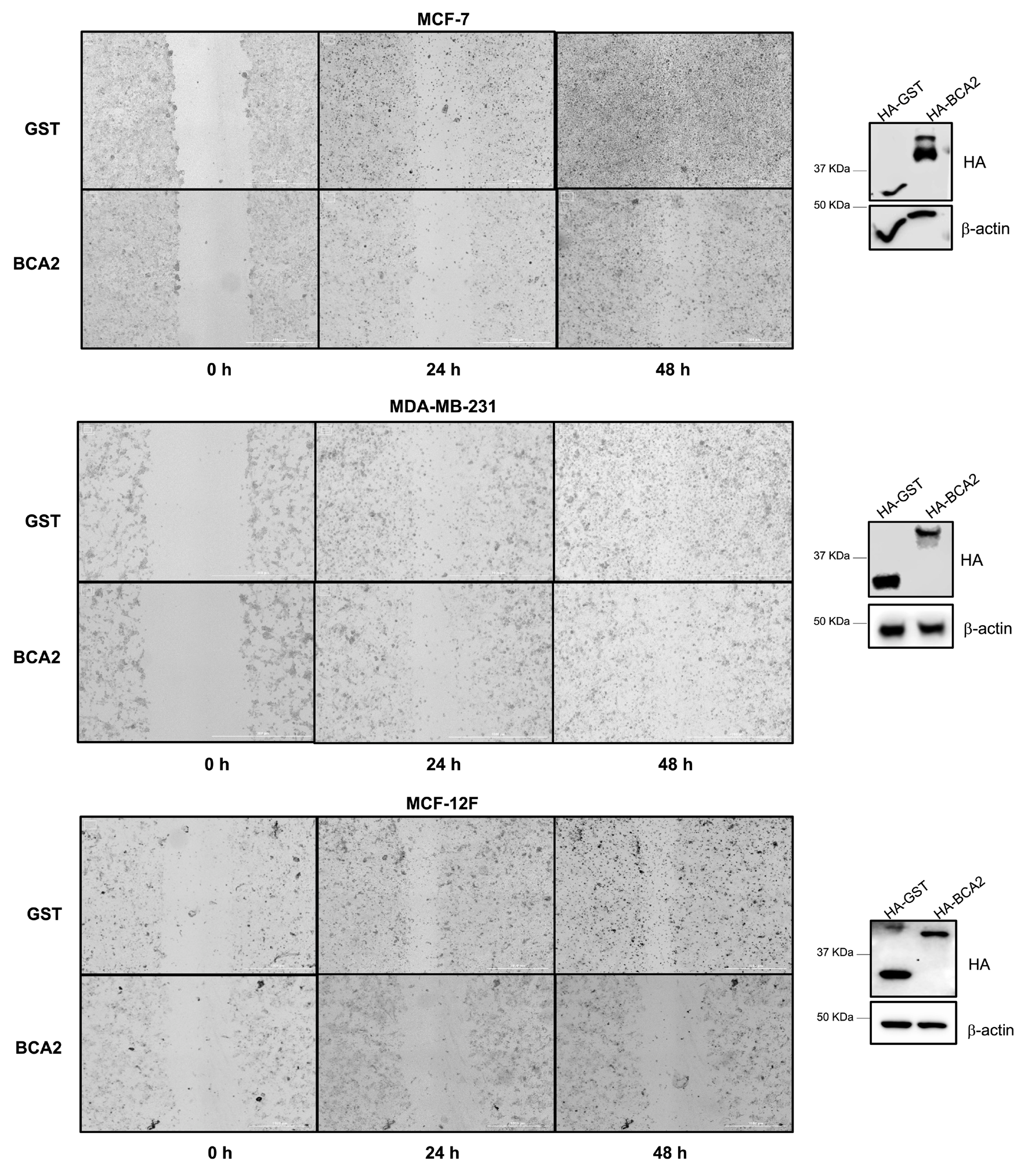

Supplement: Supplementary Figure 1 — Overexpression of BCA2 does not accelerate cell migration. Cell migration was assessed in MCF-7, MDA-MB-231, and MCF-12F cells transfected with HA-GST or HA-BCA2 over the course of 48 h. When cells reached > 90% confluence, the monolayer was scratched, and cell migration was monitored by live cell imaging. Scale bar: 1,000 μm. [file Image_1.TIFF]

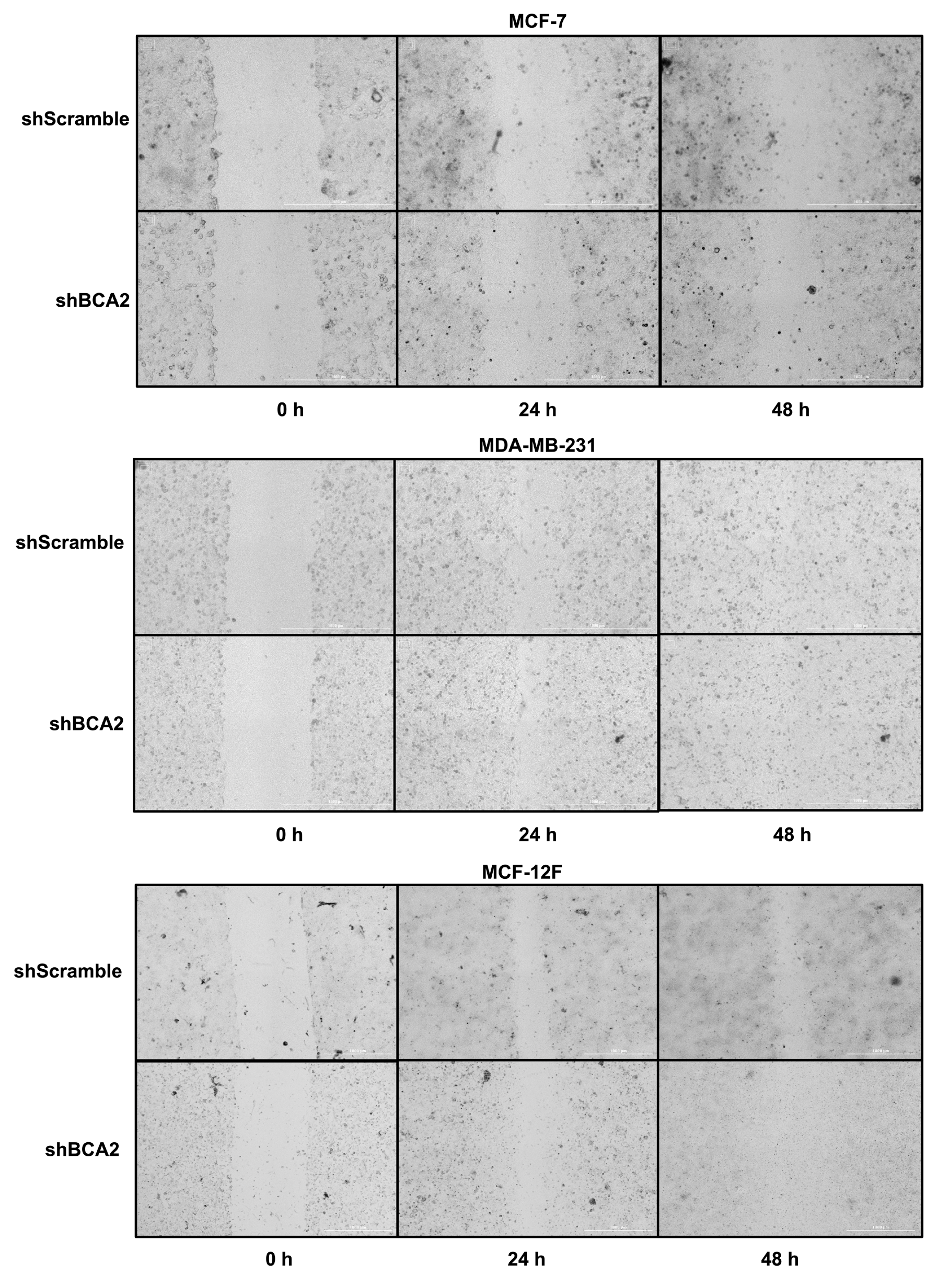

Supplement: Supplementary Figure 2 — Depletion of BCA2 delays cell migration in non-tumor breast epithelial cells. Cell migration was assessed in MCF-7, MDA-MB-231, and MCF-12F cells stably transduced with shScrambled RNA or shRNAs specific for BCA2. BCA2 knockdown was verified by RT-qPCR on the day the cells were plated (not shown). When cells reached > 90% confluence, the monolayer was scratched, and cell migration was monitored by live cell imaging. Scale bar: 1,000 μm. [file Image_2.TIFF]

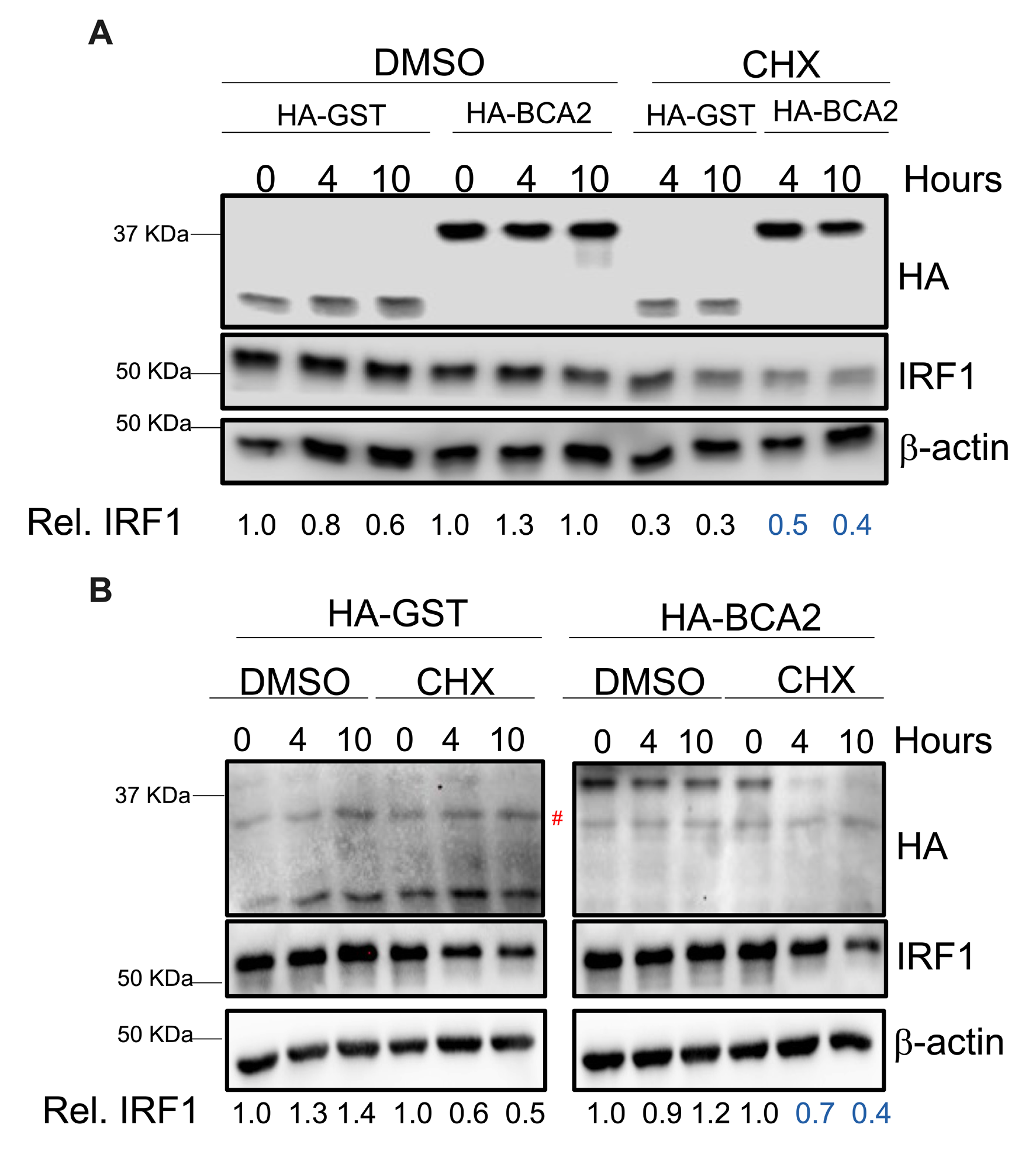

Supplement: Supplementary Figure 3 — BCA2 has no effects on IRF1 protein stability. (A,B) HEK293T cells (A) and MCF-7 cells (B) were transfected with HA-BCA2 or HA-GST (irrelevant proteins). 48 h later, cells were treated with DMSO or 200 μM of CHX for 10 h. Cells were harvested at 0, 4, and 10 h after CHX treatment. The relative levels of IRF1 are displayed underneath the blots, after correcting with β-actin levels. Blots are representative of 3 independent experiments. Red pound symbol: unspecific bands. [file Image_3.TIFF]
